# Chronic Obstructive Pulmonary Disease and COVID-19: The Impact of Hematological Biomarkers on Disease Severity and Outcomes

**DOI:** 10.3390/jcm14082765

**Published:** 2025-04-17

**Authors:** Gabriela Mara, Gheorghe Nini, Coralia Cotoraci

**Affiliations:** 1 Multidisciplinary Doctoral School, Vasile Goldis Western University of Arad, 310414 Arad, Romania; mara.gabriela@uvvg.ro; 2 Pneumology Department, Vasile Goldis Western University of Arad, 310414 Arad, Romania; nini.gheorghe@uvvg.ro; 3 Clinical Hematology Department, Vasile Goldis Western University of Arad, 310025 Arad, Romania

**Keywords:** COPD, COVID-19, hematological markers, coagulopathy, oxidative stress

## Abstract

**Background/Objectives:** Chronic obstructive pulmonary disease (COPD) patients are at heightened risk of severe COVID-19 due to underlying respiratory impairment, systemic inflammation, and immune dysregulation. This review explores the hematological changes that occur in COPD patients with COVID-19 and their implications for disease progression, prognosis, and clinical management. **Methods:** We conducted a comprehensive analysis of recent peer-reviewed studies from medical databases including Clarivate Analytics, PubMed, and Google Scholar. **Results:** Hematological alterations, such as lymphopenia, elevated neutrophil-to-lymphocyte ratio (NLR), increased D-dimer and fibrinogen levels, inflammatory anemia, and erythrocyte dysfunction, are commonly observed in COPD patients with COVID-19. These changes are linked to immune suppression, hyperinflammation, oxidative stress, and thromboembolic complications. **Conclusions:** Hematological biomarkers are valuable tools for early risk assessments and guiding treatment strategies in this high-risk population. The regular monitoring of D-dimer, fibrinogen, and NLR is advisable. Prophylactic anticoagulation and immunomodulatory therapies, such as corticosteroids and IL-6 and IL-1 inhibitors, may improve clinical outcomes. Further clinical studies are needed to validate personalized approaches and explore antioxidant-based interventions.

## 1. Introduction

Chronic obstructive pulmonary disease (COPD) is a heterogeneous lung disease marked by chronic respiratory symptoms, such as dyspnea, cough, sputum production, and exacerbations, caused by airway (e.g., bronchitis and bronchiolitis) and/or alveolar (e.g., emphysema) abnormalities that lead to persistent, often progressive, airflow obstruction [1]. The disease is primarily caused by long-term exposure to harmful particles and gases, with cigarette smoking being the leading risk factor. In 2016, an estimated 251 million cases of COPD were recorded globally, according to the Global Burden of Disease (GBD) study [2]. The Burden of Obstructive Lung Disease initiative and Global Burden of Disease collaborators have led efforts to estimate global COPD burden, reporting between 212 and 384 million cases from 2010 to 2019 based on spirometry data refined through risk factor modeling [3]. Additionally, projections from the World Health Organization (WHO) suggest that by 2030, COPD will rank as the third leading cause of death worldwide [4].

The pathogenesis of COPD involves an imbalance between proteases and antiproteases, oxidative stress, and chronic inflammation, which contribute to airway remodeling, mucus hypersecretion, and lung parenchymal destruction [5]. COPD is a major global health issue, ranking as the third leading cause of death worldwide, with an increasing burden in low- and middle-income countries [2].

Patients with COPD are at a significantly higher risk of developing severe COVID-19 infections. Pre-existing respiratory impairment and chronic inflammation in COPD patients create a favorable environment for viral infections, leading to increased disease severity and complications [6]. The presence of COPD has been associated with higher rates of hospitalization, intensive care unit (ICU) admissions, and mortality in COVID-19 patients [7]. The heightened vulnerability in COPD patients is attributed to factors such as reduced mucociliary clearance, altered immune responses, and increased expression of angiotensin-converting enzyme 2 (ACE2) receptors in the lungs, which facilitate SARS-CoV-2 entry [8].

The COVID-19 pandemic, with an estimated global excess mortality of 14.9 million in 2020 and 2021 according to WHO, has had a profound impact not only on human health but also across various fields, including epidemiology, pharmaceutical innovation, behavioral sciences, and healthcare system management [9]. It possess a greater risk of severe illness and death in individuals with underlying comorbidities, such as cardiovascular disease, diabetes, and chronic respiratory conditions. These comorbidities, along with factors like advanced age, severe lymphopenia, elevated CRP, and D-dimer levels above 1 μg/L, are strongly associated with a poor prognosis and increased mortality rates [10].

Hematological alterations play a crucial role in predicting COVID-19 severity in COPD patients. Parameters such as lymphopenia, elevated neutrophil-to-lymphocyte ratio (NLR), increased levels of C-reactive protein (CRP), and thrombocytopenia have been identified as markers of disease progression and poor outcomes [11]. In COPD patients, systemic inflammation is already elevated, which may exacerbate the hyperinflammatory state induced by COVID-19, leading to cytokine storms and severe respiratory failure [12]. Understanding these hematological changes is essential for early risk stratification, timely intervention, and improved clinical management of COPD patients with COVID-19.

The aim of this review is to investigate the hematological changes that occur in patients with COPD who are infected with COVID-19 and to explore how these changes influence disease progression, clinical outcomes, and therapeutic strategies. The review seeks to highlight the pathophysiological overlap between the two conditions, particularly in terms of immune dysregulation, coagulation abnormalities, and oxidative stress, and to evaluate the clinical utility of hematological biomarkers for risk stratification and management.

## 2. Methodology

This review is based on a comprehensive analysis of peer-reviewed studies published between January 2020 and December 2023, which were retrieved from databases including Clarivate Analytics, PubMed, and Google Scholar. Studies were selected based on their relevance to hematological abnormalities in COPD and COVID-19, particularly those addressing markers such as lymphopenia, NLR, D-dimer, fibrinogen, and indicators of oxidative stress. Both clinical and mechanistic research was included, focusing on inflammatory and coagulopathic pathways, erythropoietic dysfunction, and prognostic implications.

## 3. Shared Mechanisms Between COPD and COVID-19

### 3.1. Chronic Inflammation and Cytokine Storms

Chronic inflammation is a key pathological feature of both COPD and severe COVID-19, leading to dysregulated immune responses and systemic complications (Table 1). The interplay between chronic inflammation and hematological disturbances highlights the need for targeted anti-inflammatory and anticoagulant strategies in COPD patients at risk of severe COVID-19 complications. Understanding these mechanisms can aid in the development of personalized treatment approaches to mitigate disease progression and improve patient outcomes.

Table 1 summarizes the key inflammatory mediators and immune mechanisms involved in the cytokine storm observed in both COPD and severe COVID-19, highlighting their overlapping pathophysiological pathways.

### 3.2. Hypoxia and Oxidative Stress

COPD and severe COVID-19 are both characterized by hypoxia and oxidative stress, which contribute to systemic complications beyond lung pathology. Chronic hypoxia triggers adaptive and maladaptive responses in hematopoiesis and coagulation [13], while oxidative stress results in cellular damage and inflammation [14].

Both hypoxia and oxidative stress play a central role in the systemic complications of COPD and severe COVID-19, particularly in hematopoietic changes, immune dysregulation, and coagulation abnormalities. Understanding these mechanisms highlights the need for therapeutic strategies that target hypoxia-induced inflammation and oxidative damage, such as antioxidant therapies, oxygen supplementation, and anti-inflammatory treatments.

#### 3.2.1. Hypoxia-Induced Hematological and Coagulation Alterations in COPD and Severe COVID-19

Chronic hypoxia plays a critical role in hematopoiesis, inflammation, and coagulation dysfunction, significantly affecting disease progression in both COPD and severe COVID-19. The body’s response to persistent oxygen deprivation leads to a cascade of adaptive and maladaptive mechanisms, influencing erythropoiesis, leukocyte activity, and thrombotic risk.

Hypoxia acts as a primary stimulus for erythropoiesis, triggering the release of erythropoietin (EPO) from the kidneys to promote red blood cell (RBC) production in an effort to enhance oxygen transport, which is regulated by hypoxia-inducible factors (HIFs) [15]. In COPD, prolonged hypoxia can lead to secondary polycythemia [16], characterized by increased hematocrit levels, which may contribute to blood hyperviscosity, increased cardiovascular burden, and a higher risk of thrombotic events [17,18,19]. acute COPD exacerbations are linked to increased systemic inflammation, which promotes platelet activation, endothelial dysfunction, enhanced coagulation, and impaired fibrinolysis, as indicated by elevated platelet–monocyte complexes (PMCs), monocyte Mac-1 expression, Bon Willebrand factor (VWF), and thrombin generation, while prolonged clot lysis time and platelet hyperreactivity at admission are associated with a higher risk of relapse [20].

However, in severe COVID-19, erythropoiesis appears to be dysregulated, often leading to anemia instead of polycythemia [21]. In a study of 179 infected patients, 20.3% had a positive Coombs test (mainly IgG), though no cases of autoimmune hemolysis were found; Coombs-positive patients had more severe anemia, required more transfusions and intensive care, and showed an inflammatory anemia pattern with elevated hepcidin and ferritin but inappropriately low EPO and erythroferrone, with iron metabolism markers correlating with disease severity [22]. Consequently, while COPD patients frequently experience elevated RBC counts [23], COVID-19 patients may present with normocytic or microcytic anemia [24,25], contributing to worsened oxygen transport and disease severity.

In addition to altering RBC production, hypoxia significantly influences leukocyte function, leading to a state of chronic inflammation. Low oxygen availability enhances the release of pro-inflammatory cytokines, particularly IL-6, TNF-α, and IL-8, which promote neutrophil activation and immune cell recruitment [26]. This exaggerated inflammatory response contributes to the systemic inflammation observed in COPD and plays a role in the cytokine storm characteristic of severe COVID-19 [27].

A central regulator of this process is hypoxia-inducible factor 1-alpha (HIF-1α), a transcription factor that becomes stabilized under hypoxic conditions, leading to the upregulation of inflammatory pathways [28] by activating EGFR/PI3K/AKT pathway [29]. HIF-1α activation not only amplifies neutrophil-driven inflammation but also impairs immune resolution, resulting in sustained tissue damage and increased susceptibility to exacerbations in COPD and multi-organ failure in COVID-19 [30,31,32].

Chronic hypoxia is a well-established driver of hypercoagulability [33], predisposing patients to an increased risk of deep vein thrombosis [34,35], pulmonary embolism [36,37], and microvascular thrombosis [38,39]. This prothrombotic state is primarily mediated by

- Platelet activation and aggregation, leading to enhanced clot formation [40];
- Elevated fibrinogen levels, which contribute to increased blood viscosity [40];
- The upregulation of tissue factor (TF), a key initiator of the extrinsic coagulation cascade [41].

In COPD, chronic inflammation and endothelial dysfunction promote a hypercoagulable state, increasing the risk of cardiovascular events and stroke [42]. Similarly, in severe COVID-19, these mechanisms are further exacerbated by a viral infection, leading to the development of COVID-19-associated coagulopathy, which is strongly linked to higher mortality rates [43]. The presence of elevated D-dimer levels, along with increased fibrinogen and platelet activation, serves as a critical marker of thrombotic complications in both diseases [44].

#### 3.2.2. Increased Production of Reactive Oxygen Species (ROS) and Their Impact on Blood Cells

Oxidative stress occurs when there is an imbalance between reactive oxygen species (ROS) production and the body’s antioxidant defense, leading to cellular and tissue damage. In both COPD and severe COVID-19, excessive ROS generation plays a crucial role in disease progression, contributing to systemic inflammation, hematological alterations, and vascular dysfunction [45,46].

Several biological processes contribute to increased ROS production, further amplifying inflammation and tissue injury. Chronic hypoxia and inflammation lead to excessive mitochondrial ROS production, exacerbating oxidative stress and impairing cellular function [47]. During inflammatory responses, neutrophils generate superoxide radicals (O_2_^−^), hydrogen peroxide (H_2_O_2_), and hydroxyl radicals (OH^−^), which contribute to tissue destruction and immune dysregulation [48]. ROS reduce the availability of endothelial nitric oxide (NO), leading to vasoconstriction, impaired blood flow, and increased thrombotic risk [49].

ROS directly affect blood cell function and survival, playing a key role in hematological disturbances observed in COPD and COVID-19. Oxidative stress damages RBC membranes, leading to reduced lifespan, hemolysis, and anemia, worsening oxygen transport [50,51,52]. ROS hyperactivate neutrophils and macrophages, intensifying inflammatory responses and immune dysregulation [53,54,55]. Moreover, ROS promote platelet activation and aggregation, increasing the risk of thrombosis, microvascular injury, and multi-organ dysfunction [56,57].

The pathological effects of excessive ROS production have severe consequences in both COPD and COVID-19. In COPD, chronic oxidative stress accelerates lung tissue damage, systemic inflammation, and cardiovascular complications, leading to disease progression and frequent exacerbations [58]. In severe COVID-19, heightened ROS production is strongly associated with endothelial dysfunction, hyperinflammation (cytokine storm), and increased mortality risk [55].

Both hypoxia and oxidative stress play a central role in the systemic complications of COPD and severe COVID-19, particularly in hematopoietic changes, immune dysregulation, and coagulation abnormalities (Table 2). Understanding these mechanisms highlights the need for therapeutic strategies that target hypoxia-induced inflammation and oxidative damage, such as antioxidant therapies, oxygen supplementation, and anti-inflammatory treatments.

## 4. Hematological Changes in COPD Patients with COVID-19

Patients suffering from COPD and COVID-19 often exhibit significant hematological changes, reflecting the complex interplay between chronic inflammation, immune dysregulation, and hypercoagulability [83]. These hematological alterations contribute to disease severity, a poor prognosis, and increased risk of complications.

### 4.1. Lymphopenia and Impaired Immune Responses

Lymphopenia is a common hematological abnormality observed in COPD patients infected with SARS-CoV-2 [84]. This reduction in lymphocytes, particularly CD4+ and CD8+ T cells, impairs the host’s ability to mount an effective antiviral immune response [85,86]. CD4+ T cells play a crucial role in orchestrating adaptive immunity by activating B cells and cytotoxic T cells, while CD8+ T cells are essential for the direct elimination of virus-infected cells [87]. The depletion of these immune cells weakens the body’s defense mechanisms, allowing for uncontrolled viral replication and prolonged infection [88].

Another critical marker of immune dysfunction in COPD patients with COVID-19 is an increased NLR. A high NLR is indicative of an exaggerated inflammatory response and has been established as a reliable prognostic marker for severe disease progression and mortality [89]. The combination of lymphopenia and elevated neutrophil counts promotes systemic inflammation, tissue damage, and multi-organ failure, exacerbating the already compromised pulmonary function in COPD patients [90].

### 4.2. Coagulopathy and Thromboembolic Risk

Coagulopathy is a significant concern in COPD patients with COVID-19 due to the heightened inflammatory state and endothelial dysfunction associated with both conditions. Elevated levels of D-dimer, fibrinogen, and vWF have been reported in these patients, reflecting an increased propensity for thrombotic complications. D-dimer, a fibrin degradation product, serves as a biomarker of hypercoagulability and is frequently elevated in severe COVID-19 cases, correlating with a higher risk of adverse outcomes [91].

One of the most concerning thromboembolic complications in COPD patients with COVID-19 is pulmonary embolism (PE) [92]. The chronic hypoxia and systemic inflammation present in COPD contribute to a prothrombotic environment, which, when compounded by COVID-19, significantly increases the risk of PE [83]. Furthermore, the presence of disseminated intravascular coagulation has been observed in critically ill COVID-19 patients, manifesting as widespread microvascular thrombosis and the consumption of clotting factors, leading to life-threatening hemorrhagic complications [93]. These hematological alterations necessitate close monitoring and early intervention with anticoagulant therapy to mitigate thrombotic risks.

### 4.3. Inflammatory Anemia and Erythrocyte Dysfunction

Chronic hypoxia in COPD patients leads to compensatory erythropoiesis, often resulting in secondary polycythemia. However, in the context of COVID-19, systemic inflammation disrupts normal erythropoiesis, leading to inflammatory anemia (also known as anemia of chronic disease) [94,95,96]. Pro-inflammatory cytokines such as IL-6 and TNF-α interfere with iron metabolism by increasing hepcidin levels, which inhibits iron absorption and sequestration, thus impairing hemoglobin synthesis [97].

Additionally, erythrocyte dysfunction is a notable concern in COPD patients with COVID-19. Oxidative stress and systemic inflammation contribute to altered erythrocyte membrane properties, reducing their deformability and increasing their susceptibility to hemolysis [98]. This leads to impaired oxygen delivery to tissues, further exacerbating hypoxia and worsening disease outcomes [99]. The combination of chronic hypoxia, inflammation-driven iron deficiency, and erythrocyte dysfunction highlights the complex hematological challenges faced by COPD patients during COVID-19 infection [100,101].

**Figure 1:**
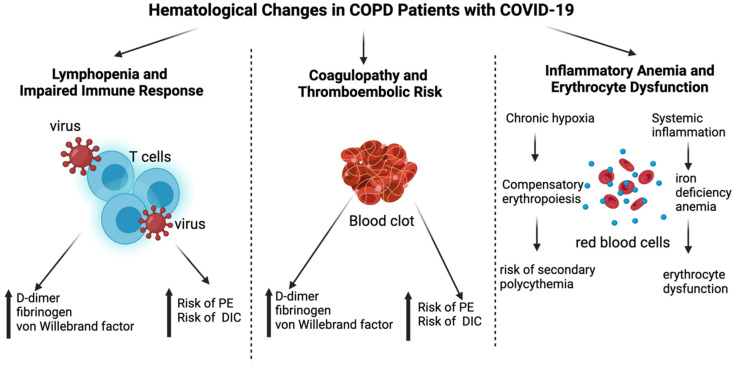
Hematological changes in COPD patients with COVID-19. This figure illustrates the key hematological changes observed in COPD patients with COVID-19. The three main categories included are as follows: 1. Lymphopenia and Impaired Immune Response—Characterized by a reduction in CD4+ and CD8+ T cells, leading to weakened antiviral defense, and an increased neutrophil-to-lymphocyte ratio (NLR), which serves as a prognostic marker; 2. Coagulopathy and Thromboembolic Risk—Marked by elevated D-dimer, fibrinogen, and von Willebrand factor levels, increasing the risk of pulmonary embolism (PE) and disseminated intravascular coagulation (DIC); 3. Inflammatory Anemia and Erythrocyte Dysfunction—Chronic hypoxia induces compensatory erythropoiesis, increasing the risk of secondary polycythemia, while systemic inflammation contributes to iron deficiency anemia and erythrocyte dysfunction. Arrows indicate relationships between the central concept and its specific pathological mechanisms. Created with Biorender.com.

Figure 1 highlights the hematological changes in COPD patients with COVID-19, focusing on three main aspects: lymphopenia and impaired immune response, coagulopathy and thromboembolic risk, as well as inflammatory anemia and erythrocyte dysfunction, each contributing to disease severity and patient prognosis.

## 5. Clinical Implications and Therapeutic Strategies

### 5.1. Monitoring Hematological Markers

The management of COPD patients infected with COVID-19 necessitates the diligent monitoring of hematological markers, which act as crucial prognostic indicators. Among these markers, D-dimer, fibrinogen, and the neutrophil-to-lymphocyte ratio (NLR) are particularly significant in assessing disease severity and predicting adverse outcomes [102].

The role of hematological markers in prognosis is significant in various medical conditions. Elevated D-dimer levels are strongly associated with an increased risk of thrombotic complications, particularly in COVID-19 patients, and can serve as an early indicator of coagulopathy [103]. High fibrinogen levels reflect an ongoing inflammatory response and a heightened risk of clot formation, both of which are crucial concerns in COPD patients with COVID-19 [104]. Additionally, a high neutrophil-to-lymphocyte ratio (NLR) is indicative of an exaggerated inflammatory response and is correlated with worse clinical outcomes in COVID-19 [105].

Given the prognostic significance of these hematological markers, screening algorithms for COPD patients with COVID-19 should incorporate routine monitoring of D-dimer, fibrinogen, and NLR. The early identification of abnormalities in these markers can help clinicians stratify patients based on risk levels and tailor interventions accordingly.

The table below outlines essential hematological monitoring strategies used in the clinical management of COPD patients with COVID-19, emphasizing their diagnostic and prognostic value, along with associated benefits and limitations (Table 3).

In clinical practice, hematological monitoring should begin at hospital admission for all COPD patients diagnosed with COVID-19. For hospitalized patients, D-dimer, fibrinogen, and NLR should be assessed on admission [114]. Suggested thresholds to consider therapeutic escalation include:

- D-dimer > 1.0 µg/mL, which is associated with increased thrombotic risk and may justify the initiation or intensification of anticoagulation;
- NLR > 7, which is predictive of severe disease progression and a poor prognosis;
- Fibrinogen > 4 g/L, which is indicative of high inflammatory burden.

These values should be interpreted along with clinical status, oxygenation levels, and imaging findings. Individualized management strategies based on dynamic changes in these markers can support timely intervention and improved outcomes.

### 5.2. Anticoagulation Therapy in COPD and COVID-19

Prophylactic anticoagulation plays a crucial role in preventing thromboembolic complications, particularly in patients with COPD and COVID-19, who are already prone to clot formation due to their compromised respiratory and circulatory systems. Given these risks, prophylactic anticoagulation is strongly recommended as a preventative measure to reduce morbidity and mortality in affected patients [115].

The primary benefits of prophylactic anticoagulation include the prevention of deep vein thrombosis (DVT) and pulmonary embolism (PE), which are common complications in severe COVID-19 cases, often leading to worsened respiratory function and increased mortality rates [116]. By inhibiting excessive clotting, anticoagulation significantly reduces the likelihood of these life-threatening events. Another key benefit is the reduction in mortality and morbidity. Patients receiving anticoagulation therapy show lower rates of thrombotic complications, ultimately improving their overall prognosis. By preventing clot formation, these therapies contribute to better systemic oxygenation and reduced strain on the cardiovascular system, thereby enhancing recovery outcomes.

Several anticoagulants are available for prophylactic use, each with specific advantages and considerations for patients with COPD and COVID-19.

Low-molecular-weight heparin (LMWH) is a class of anticoagulants and is often the preferred choice due to its predictable pharmacokinetics and lower risk of bleeding compared to unfractionated heparin [117]. LMWH is administered via subcutaneous injection and does not require frequent laboratory monitoring, making it a practical option for hospitalized and critically ill patients.

Direct oral anticoagulants (DOACs) are anticoagulants which offer an alternative to LMWH and are particularly useful for long-term management. However, their use requires careful dose adjustments in patients with renal impairment, which is a common comorbidity in individuals with COPD [118]. While DOACs provide convenience through oral administration, their safety profile necessitates close monitoring, especially in those with fluctuating renal function [119]. Studies have shown that direct oral anticoagulants (DOACs), such as apixaban, rivaroxaban, dabigatran, and edoxaban, offer superior efficacy and safety compared to traditional oral anticoagulant (OAC) therapies, including vitamin K antagonists (VKAs), in patients with pulmonary disease [120].

Additional clinical considerations should be given to COPD patients with coexisting COVID-19 and renal impairment. Renal dysfunction can significantly alter the pharmacokinetics of anticoagulants, particularly DOACs, increasing the risk of bleeding or subtherapeutic dosing. In this context, the regular monitoring of renal function (e.g., serum creatinine and eGFR) is essential to guide dose adjustments or the choice of anticoagulant type [121].

Although DOACs and LMWH are generally preferred, vitamin K antagonists (VKAs) such as warfarin remain in use for certain patient populations, particularly those with mechanical heart valves, antiphospholipid syndrome, or severe renal impairment, where DOACs may be contraindicated. VKAs require regular INR monitoring but may be a practical option in settings where other anticoagulants are not available or are cost-prohibitive [122].

Table 4 presents a comparative summary of anticoagulant options commonly used in COPD patients with COVID-19, detailing their mechanisms of action, clinical benefits, and associated risks to support individualized treatment decisions.

In conclusion, prophylactic anticoagulation is a critical intervention in managing patients with COPD and COVID-19. By mitigating the risks associated with thromboembolic events, these treatments contribute significantly to improving patient outcomes, reducing complications, and ultimately enhancing survival rates in this vulnerable population. Further research and clinical guidelines will continue to refine the best practices for anticoagulation strategies, ensuring optimal care for high-risk patients.

### 5.3. Immunomodulators and Anti-Inflammatory Therapy

Inflammatory responses play a crucial role in the pathophysiology of COVID-19-associated respiratory deterioration, particularly in COPD patients who already have compromised pulmonary function. Immunomodulatory therapies aim to control cytokine storms and excessive inflammation [129].

Corticosteroids, such as dexamethasone, are widely used in COVID-19 management due to their ability to reduce lung inflammation and lower mortality in severe cases. Their benefits include reducing cytokine-mediated lung damage, improving oxygenation, and decreasing the need for mechanical ventilation [130]. However, they also carry certain risks, such as immune suppression, which can increase susceptibility to secondary infections and worsen COPD exacerbations [131].

Severe COVID-19 can lead to a cytokine storm, which exacerbates lung injury. Monoclonal antibodies such as Tocilizumab (IL-6 inhibitor) and Anakinra (IL-1 receptor antagonist) have been investigated for their role in mitigating excessive immune responses [132].

Another emerging strategy involves specialized pro-resolving mediators (SPMs), such as resolvins, protectins, and maresins, which actively promote the resolution phase of inflammation without inducing immunosuppression [133]. SPMs have shown potential in preclinical models for reducing cytokine-driven damage and restoring immune homeostasis [133], which could be particularly beneficial in COPD patients with COVID-19 experiencing excessive inflammatory responses.

Table 5 outlines key immunomodulatory agents used in COPD patients with COVID-19, focusing on their mechanisms of action, therapeutic benefits, and potential risks, with a particular emphasis on cytokine-targeted strategies.

In addition to acute management, attention must be given to the long-term consequences of SARS-CoV-2 infection in COPD patients. Recent studies have highlighted that individuals with COPD are particularly prone to developing post-COVID-19 syndrome, often characterized by persistent fatigue, dyspnea, and neuropsychiatric symptoms. In a cohort study conducted in Romania, symptoms such as fatigue (36%), cough (26%), and myalgia (23%) were observed up to six months post-infection, especially in those with pre-existing respiratory conditions and comorbidities [139]. These findings underscore the importance of integrated post-discharge care and tailored rehabilitation for COPD patients recovering from COVID-19.

## 6. Conclusions

Patients with chronic obstructive pulmonary disease (COPD) represent a high-risk population during the COVID-19 pandemic due to their underlying chronic inflammation, persistent hypoxia, and immune dysregulation. The presence of SARS-CoV-2 infection further exacerbates these conditions by triggering an intense systemic inflammatory response, which is closely associated with significant hematological changes.

Common hematological abnormalities in COPD patients with COVID-19, such as lymphopenia, elevated NLR, increased levels of D-dimer and fibrinogen, inflammatory anemia, and erythrocyte dysfunction, have been strongly correlated with disease severity, a poor prognosis, and a higher risk of thromboembolic complications. These alterations serve not only as reflections of disease progression but also as important prognostic markers that can guide early clinical intervention.

Chronic hypoxia and oxidative stress, which are hallmark features of both COPD and severe COVID-19, amplify inflammatory and thrombotic responses through mechanisms that involve HIFs and the excessive production of reactive oxygen species (ROS). These processes contribute to systemic damage, cellular dysfunction, and vascular complications that significantly impact patient outcomes.

From a clinical perspective, the continuous monitoring of hematological markers, particularly D-dimer, fibrinogen, and NLR, is crucial for timely risk stratification and therapeutic decision-making. Prophylactic anticoagulation-tailored anti-inflammatory therapies and immunomodulators have emerged as key strategies in managing these patients, and their use should be personalized based on individual hematological profiles and comorbid conditions.

Future research should focus on evaluating the efficacy and safety of anticoagulants and immunomodulatory treatments specifically in COPD patients with COVID-19. Additionally, clinical guidelines should emphasize personalized approaches that integrate hematological assessments as core components of care. Investigating the therapeutic potential of antioxidant strategies and targeted interventions to reduce oxidative stress may further improve clinical outcomes in this vulnerable population.

## Figures and Tables

**Table 1:** Chronic inflammation and cytokine storms in COPD and severe COVID-19.

Mechanism	Description	Relevance to COPD	Relevance to Severe COVID-19
Increased IL-6 levels	IL-6 is a key pro-inflammatory cytokine involved in immune activation.	Elevated in COPD, especially in frequent exacerbators; associated with reduced lung function.	Correlated with disease severity and cytokine storms; higher serum levels linked to poor outcomes.
Elevated TNF-α levels	Promotes macrophage activation and lung tissue damage.	Increased in COPD; contributes to alveolar destruction and exacerbations.	Associated with ARDS and lung injury; a potential target for anti-TNF therapies.
Increased IL-8 (CXCL8) expression	Recruits neutrophils and amplifies inflammation.	Associated with exacerbations and lung function decline.	Enhances neutrophil infiltration, worsening alveolar injury.
Macrophage and dendritic cell activation	Innate immune cells regulate inflammatory responses.	Overactive macrophages produce cytokines and sustain inflammation.	Excessive activation linked to cytokine storms and multiorgan failure.
NET formation and neutrophilia	Neutrophils release extracellular traps that damage tissue.	Higher levels correlate with worse symptoms and exacerbation frequency.	Associated with disease severity and increased cfDNA and mortality.
Hematological alterations and coagulopathy	Inflammation affects coagulation and increases thrombosis risk.	Advanced COPD increases VTE risk and worsens the prognosis if thrombotic events occur.	Coagulopathy may result in pulmonary embolism and stroke; associated with increased mortality.
Oxidative stress and lung damage	ROS generation exacerbates inflammation and tissue injury.	Drives COPD progression and systemic inflammation.	Contributes to endothelial injury, pulmonary fibrosis, and cytokine-mediated damage.

**Table 2:** Biomarkers of hypoxia and oxidative stress in COPD and severe COVID-19.

Biomarker	Description	Relevance in COPD	Relevance in Severe COVID-19
Erythropoietin (EPO)	Hormone produced by the kidneys to stimulate red blood cell (RBC) production in response to hypoxia.	Increased due to chronic hypoxia, leading to secondary polycythemia [59]	Variable: May be low or normal due to inflammation-related erythropoiesis [60,61]
Hemoglobin (Hb) and Hematocrit (Hct)	Measures of oxygen-carrying capacity in blood.	Increased in some COPD patients due to chronic hypoxia [62]	Decreased in severe COVID-19, often due to anemia of inflammation [63]
Hypoxia-Inducible Factor 1-alpha (HIF-1α)	A transcription factor activated by low oxygen levels; regulates cellular responses to hypoxia.	Upregulated, promoting erythropoiesis, angiogenesis, and glycolysis [64].	Highly upregulated, contributing to immune activation, inflammation, and vascular remodeling [65].
Reactive Oxygen Species (ROS)	Free radicals produced by mitochondria and immune cells during oxidative stress.	Increased, leading to oxidative damage in lung tissue [45].	Increased, contributing to endothelial dysfunction and tissue injury [66].
Malondialdehyde (MDA)	Malondialdehyde (MDA)	Elevated, reflecting cell membrane damage due to chronic inflammation [67].	Elevated, linked to severe oxidative injury in the lung and vascular endothelium [68].
Superoxide Dismutase (SOD)	An antioxidant enzyme that neutralizes superoxide radicals.	Increased, leading to higher oxidative stress and lung injury [69].	Decreased, reducing protection against inflammation-induced ROS [70].
Glutathione (GSH)	A major intracellular antioxidant that protects against oxidative damage.	Depleted, leading to chronic lung tissue damage [71].	Depleted, impairing the immune response and increasing lung injury risk [72].
8-Hydroxy-2′-deoxyguanosine (8-OHdG)	A DNA oxidation marker, indicating oxidative DNA damage.	Increased, reflecting oxidative stress-induced genetic damage in lung cells [73].	Increased, linked to severe systemic inflammation and endothelial dysfunction [74].
Fibrinogen	An acute-phase protein involved in blood clot formation.	Elevated, contributing to hypercoagulability and increased thrombosis risk [75].	Highly elevated, associated with coagulation disorders and microthrombosis [76].
D-dimer	A fibrin degradation product and a marker of clot formation and breakdown.	Mildly elevated, especially in exacerbations or comorbid cardiovascular disease [77].	Significantly elevated, indicating high thrombotic activity and a poor prognosis [78].
Interleukin-6 (IL-6)	A pro-inflammatory cytokine involved in immune regulation and inflammation.	Increased, associated with disease severity and systemic inflammation [79].	Markedly increased, linked to cytokine storms and respiratory failure [80].
Myeloperoxidase (MPO)	An enzyme released by neutrophils and a marker of oxidative stress and inflammation.	Elevated, contributing to neutrophil-mediated lung damage [81].	Elevated, associated with endothelial dysfunction and multi-organ injury [82].

**Table 3:** Key hematological monitoring strategies in COPD patients with COVID-19.

Therapeutic Strategy	Purpose	Advantages	Risks/Limitations	References
D-dimer monitoring	Assess thrombotic risk and predict severity	Early detection of coagulopathy and guides anticoagulation	False positives must be interpreted with other markers	[106,107,108]
Fibrinogen monitoring	Evaluate inflammation and coagulation status	Helps in risk stratification	Can be elevated due to multiple factors that are not always specific to COVID-19	[109,110]
NLR assessment	Gauge severity of inflammation	Quick and cost-effective	Non-specific, influenced by other infections or stress responses	[111,112,113]

**Table 4:** Comparative overview of anticoagulant therapies in COPD patients with COVID-19.

Anticoagulant Type	Mechanism	Benefits	Risks/Limitations	References
LMWH	Inhibits factor Xa and prevents clot formation	Reliable dosing; fewer drug interactions	Requires injection and costlier than DOACs	[123,124,125]
DOACs	Directly inhibit factor Xa or thrombin	Oral administration; no need for monitoring	Bleeding risk; contraindications in renal impairment	[125,126,127]
VKAs (e.g., warfarin)	Inhibits vitamin K-dependent clotting factors (II, VII, IX, X)	Effective for patients with mechanical valves or antiphospholipid syndrome; cost-effective	Requires regular INR monitoring; dietary restrictions; drug interactions	[122,128]

**Table 5:** Overview of immunomodulatory therapies in the management of COPD patients with COVID-19.

Immunomodulator	Mechanism	Benefits	Risks/Limitations	References
Corticosteroids	Reduce inflammatory cytokine release	Lower mortality and improved oxygenation	Increased infection risk and hyperglycemia	[134]
Tocilizumab	An IL-6 receptor blocker; suppresses inflammation	Reduces cytokine storm severity	May cause hepatotoxicity and secondary infections	[135,136]
Anakinra	Blocks IL-1 receptor and mitigates hyperinflammation	Beneficial in severe cases	Limited evidence and high cost	[137,138]
